# Modeling Transmission Dynamics and Control of Vector-Borne Neglected Tropical Diseases

**DOI:** 10.1371/journal.pntd.0000761

**Published:** 2010-10-26

**Authors:** Paula M. Luz, Claudio J. Struchiner, Alison P. Galvani

**Affiliations:** 1 School of Public Health, Yale University, New Haven, Connecticut, United States of America; 2 Instituto de Pesquisa Clinica Evandro Chagas, Fundacao Oswaldo Cruz, Manguinhos, Rio de Janeiro, Rio de Janeiro, Brasil; 3 Programa de Computacao Cientifica, Fundacao Oswaldo Cruz, Manguinhos, Rio de Janeiro, Rio de Janeiro, Brasil; London School of Hygiene & Tropical Medicine, United Kingdom

## Abstract

Neglected tropical diseases affect more than one billion people worldwide. The populations most impacted by such diseases are typically the most resource-limited. Mathematical modeling of disease transmission and cost-effectiveness analyses can play a central role in maximizing the utility of limited resources for neglected tropical diseases. We review the contributions that mathematical modeling has made to optimizing intervention strategies of vector-borne neglected diseases. We propose directions forward in the modeling of these diseases, including integrating new knowledge of vector and pathogen ecology, incorporating evolutionary responses to interventions, and expanding the scope of sensitivity analysis in order to achieve robust results.

## Introduction

Mathematical modeling of vector-borne infectious diseases originated with Sir Ronald Ross's study of malaria transmission in 1916 [1]. Ross recognized that vector-borne infections are governed by nonlinear dynamics, which makes intuitive assessment of the natural trajectory of an epidemic and intervention effectiveness difficult, if not impossible, without mathematical modeling. Mathematical models can play important roles in the study of infectious diseases. Models help explain the dynamics of an infectious disease within a host or a population, and they facilitate comparisons among competing control strategies that can inform policy decisions.

The use of mathematical models has been gaining momentum in recent decades. Models are being used to address an ever-expanding number of diseases and public health questions, as well as to explore the importance of biological and ecological details on disease transmission [2]. For example, to realistically incorporate the population dynamics of mosquitoes, there is a need to take into account age structure, seasonality, and density-dependent mortality [3]–[5]. The realistic incorporation of vectors then improves the evaluation of the long-term impact of control strategies [6], [7]. In this review, we address model parameterization and sensitivity analysis, two important steps in the building and analysis of mathematical models. We discuss specific features of neglected vector-borne diseases that should be incorporated into quantitative methods that analyze control strategies for such diseases. We also review the areas in which models have been and can be most useful, including drug, vaccine, vector, and alternative control strategies, as well as cost-effectiveness analysis.

## Methods

We searched PubMed, Web of Science, and SciELO using the terms “mathematical model”, “modeling”, “cost-effectiveness analysis”, and “economic analysis”. For the theoretical literature, we included studies that, in our opinion, addressed important technicalities of mathematical models applied to infectious diseases, including, for example, dynamic modeling, sensitivity analysis, and cost-effectiveness analysis. Modeling analyses conducted for diseases not considered to be neglected vector-borne diseases were excluded. We report on studies that evaluate the impact of interventions on vector-borne neglected tropical diseases.

## Basic Model Development, Parameterization, and Sensitivity Analyses

**Figure 1:**
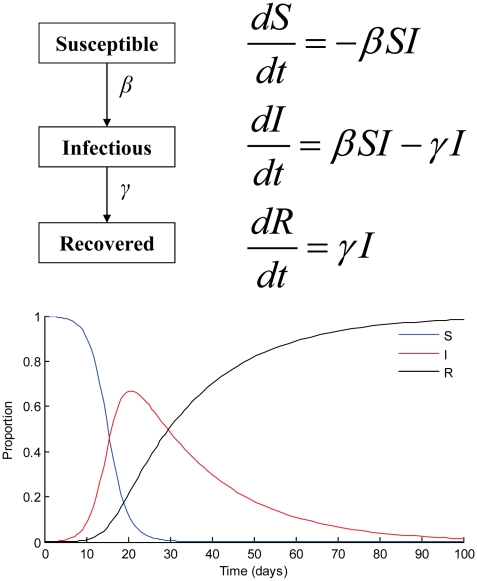
SIR model. Schematic representation, differential equations, and plot for the basic SIR (susceptible, infectious, and recovered) model. Model parameters are *β*, the transmission rate (*β* = 0.0005), and *γ*, the recovery rate (*γ* = 0.05). There is initially one infection in a population of 1,000 individuals.

Model development involves several steps and considerations. Once the modeler identifies the essential components of the biological processes necessary to address the questions of interest, the information needs to be translated into equations that describe the transmission dynamics. The most popular mathematical model is the SIR model, which divides hosts into compartments on the basis of whether they are susceptible, infectious, or recovered/immune (Figure 1). A susceptible individual (*S*) who contracts disease becomes infectious (*I*) and then recovers (*R*) to become immune. The parameters of the SIR model are the rate at which susceptible hosts become infected (*β*) and the rate at which infectious individuals recover (*γ*). For vector-borne diseases, the rate at which hosts become infected (*β*) depends on vector competence and abundance.

Model parameterization can be achieved using published studies and from fitting a model to observed data [2]. As parameter values are only estimates of “true” values [8], modelers need to perform sensitivity analysis in order to explore which parameters have the greatest impact on model predictions. Different approaches to sensitivity analysis vary in their simplicity and applicability to specific models. Univariate sensitivity analysis measures the impact of the variation of one parameter on the outcome of the model while all other parameters are held constant. One method for conducting univariate sensitivity analysis is to change the value of each model parameter by a certain percent, and then measure the percent change in the value of the outcome. Such sensitivity analysis can be represented on a tornado plot, which is a graphical way of showing which parameters most strongly influence the outcome of a model [9]. For vector-borne diseases, it is common that demographic parameters, such as the vector's life span and reproductive capacity, have the greatest impact on the population dynamics of the vector. For example, the mortality rate of a vector usually has a particularly pronounced influence on disease transmission [10].

Multivariate sensitivity analysis consists of simultaneously measuring the impact of multiple parameters. One approach for multivariate sensitivity analysis is through Monte Carlo simulations. For this procedure, probability distributions are assigned to parameters and the values of those parameters are sampled repeatedly from these distributions. Model simulations with each set of these parameters are then computed to generate a distribution of model outcomes from which summary statistics can be calculated. Statistical regression models can then be used to determine which parameters most strongly influence model outcome [11].

## Current Public Health Challenges Addressed by Models of Vector-Borne Diseases

Mathematical models and cost-effectiveness analysis have been used to assess the impact of various control strategies for a wide range of neglected tropical diseases, which we review here.

### Maximizing Drug Utility

Many neglected vector-borne diseases can be treated and controlled with drugs [12]. However, this control strategy imposes selection for drug resistance [13], [14]. To maximize long-term drug utility, such evolutionary consequences should be taken into account. Currently, several vector control programs advocate widespread administration of drugs to avoid mass screening for detection of infected individuals, because diagnostic tests can be costly and imperfect [15]. However, mass administration of drugs results in the unnecessary treatment of uninfected individuals, a practice leading to higher rates of adverse effects and faster selection for drug resistance [16]. For example, the dilemma of mass versus targeted drug administration for onchocerciasis, a disease usually treated with the drug ivermectin, has been explored using a model that incorporates heterogeneity in human exposure [16]. It was found that targeted ivermectin interventions can reduce the onchocerciasis health burden using only 20%–25% of the doses required for mass drug administration, thus resulting in decreased costs, a smaller proportion of adverse effects, and a lower probability of spread of ivermectin resistance [16]. This example illustrates the positive impact on treatment approaches that modeling public health interventions can have to reduce both the spread of disease and the development of resistance.

### Vector Control

Vector control [12], which relies on the use of insecticides, is the primary control method of neglected vector-borne diseases. The basic reproduction number (*R*_0_) is the number of secondary infections generated from a single infected individual introduced into a susceptible population. In order to curtail transmission, vector control efforts need to decrease the value of *R*_0_ below the critical value of 1. For example, *R*_0_ was used to determine the extent of vector control necessary to eliminate the transmission of Chagas disease in Brazil [17]. Given that the *R*_0_ for Chagas disease in Brazil is 1.25, it was shown that a 25% increase in vector control mortality induced by insecticides was sufficient to reduce *R*_0_ below 1. Nonetheless, a differential equation model showed that a vector control strategy that reduced *R*_0_ just below 1 would require more than half a century to achieve disease eradication due to disease persistence in chronically infected individuals.

Two models incorporating vector control have also evaluated insecticide-based vector control strategies for dengue prevention [18], [19]. The estimated impact of vector control on dengue cases differs between the two studies due to different model assumptions regarding the seasonality of dengue transmission. When seasonality is not incorporated, control of adult mosquitoes is predicted to delay but not eliminate dengue epidemics. Thus, ultimately, vector control is predicted to have little impact on dengue incidence [19]. In contrast, when seasonality is incorporated, control of adult mosquitoes is found to be the most effective strategy to curtail an ongoing epidemic [18]. This highlights the general phenomenon that the omission of fundamental biological realism can significantly affect model predictions.

Field evaluations have shown that resistance evolution currently threatens dengue vector control strategies [20]. A mathematical model of seasonal *Aedes aegypti* population dynamics that incorporates population genetics has been used [21] to estimate the impact of insecticide-based vector control interventions. Considering both the impact of interventions on mosquito abundance and on the evolutionary trajectory of resistance, it was found that optimal interventions combine vector control at both larval and adult stages and are applied only during the dengue season.

### Vaccine Delivery and Coverage

One of the most significant obstacles to the implementation of optimal vaccination policies is public adherence to recommendations. Concerns about risks of vaccinations, whether real or perceived [22]–[25], affect vaccination decisions. A burgeoning area for the application of models has been the prediction of likely adherence to different recommendations, particularly with regard to designing strategies to promote optimal vaccination recommendations. Models can be used to analyze both public health policy and individual perspectives on a vaccination policy, such as in the case of mosquito-borne yellow fever [26], [27]. Sylvatic yellow fever affects most of the north and central-west regions of Brazil. The coastal area of Brazil, where the majority of the population lives, is infested with the vector that transmits urban yellow fever, posing a risk of an urban yellow fever outbreak. In recent years, several reports of yellow fever vaccine-related adverse events have generated public concern about the safety of the vaccine [28]. The risk of a vaccine-related adverse fatal event versus the risk of an urban outbreak poses a dilemma for vaccination policies in areas to which yellow fever has not yet spread.

When the risk of serious adverse events from vaccination and infection were taken into account, actual vaccination levels were predicted to be lower than the vaccination levels required to prevent an outbreak [27]. From the individual perspective, the decision of whether to vaccinate becomes a function of how prepared the public health authorities are for an urban outbreak [26]. If preparedness is high, the optimal strategy for the individual is to wait for an outbreak to actually occur before getting vaccinated. However, the choice to vaccinate before an outbreak becomes a better strategy as the likelihood of an outbreak increases [26].

### New and Alternative Interventions

Models can be used to evaluate the benefit of innovative control strategies before devoting resources to the actual development and implementation. For example, a mathematical model of onchocerciasis transmission was used to evaluate the impact of a hypothetical macrofilaricidal drug [29]. It was predicted that the hypothetical drug would increase the potential for onchocerciasis elimination compared to ivermectin, the drug currently in use. However, depending on the epidemiological scenario of a particular setting, high treatment coverage would still be needed [29].

Other studies have assessed the potential impact of zooprophylaxis interventions, that is, the use of animals resistant against disease to divert bites from humans, with regard to the household transmission of Chagas disease [30]. The model considers the vector, other domestic animals, and age structure in the human population within the household. It was found that increasing the domiciliary chicken population would not impact human prevalence rates significantly. Conversely, the exclusion of other infectious vertebrates, especially infected dogs, from the domestic environment can effectively reduce the human prevalence rate [30].

Another promising avenue for the control of vector-borne disease is through the genetic manipulation of vectors, an approach that could be used synergistically with current control strategies [31]. Genetic methods for controlling vector transmission are designed to reduce or eliminate vector populations, to selectively kill only infected vectors, or to modify (or replace) natural vector populations by introgressing genes that hamper vector competence.

Transgenic strategies can be categorized as strategies that block transmission, either from humans to mosquitoes or from mosquitoes to humans; strategies that reduce mosquito biting by interfering with host-seeking behavior; strategies that raise overall mosquito mortality, i.e., through the release of engineered males homozygous for a dominant female-killing gene; or strategies that raise mosquito infection-induced mortality, i.e., lethal genes only expressed in the presence of infection. The evolutionary impact of these different transgenic strategies must be incorporated to fully evaluate the benefits, risks, and research priorities associated with using genetically manipulated insects to control vector-borne diseases [32]. Despite their promise as new tools to reduce disease transmission, these interventions select for changes in pathogen virulence to both the human and mosquito hosts, and their evolutionary impact remains to be explored. Modeling has shown that transgenic strategies based on blocking transmission or reducing mosquito biting could select for increased disease virulence to humans [33]. By contrast, strategies that increase mosquito mortality do not select for changes in virulence to humans [33].

### Combined Interventions

Given that pathogens rapidly evolve to evade interventions, the greatest promise for successful long-term control of vector-borne disease may be a combined approach. The optimal combination of control strategies can be assessed with mathematical models. The dynamic aspect of all infectious diseases lends itself to adaptive responses, which may translate into different optimal combinations of interventions in different locations or at different times.

A modeling study evaluated the duration of mass treatment, drug coverage, the added benefit of vector control, and the possibility of resistance to drugs used in the mass drug administration program for the control of lymphatic filariasis [34]. It was found that in areas where the disease is highly endemic, adding vector control to the mass drug administration program greatly increases the speed at which control is attained. There are synergistic benefits of using both mass drug administration and vector control, because the former affects current infections while the latter prevents new infections [34].

A more complex model incorporating host age-structured and vector transmission dynamics was used to estimate the impact of intervention strategies that consider two community-based interventions for filariasis control, vector control, and/or single-dose mass chemotherapy [35]. Vector control was simulated by reducing the biting rate, leading to a gradual decrease in human infection levels. By incorporating host age-structure, the impact of vector control on averting infections in the youngest age groups was estimated. Chemotherapeutic interventions were predicted to reduce both the prevalence and intensity of infection. After cessation of treatment, recovery of infection levels depended on the anti-filarial drug used, the two most common being diethylcarbamazine citrate and ivermectin. Diethylcarbamazine citrate, which kills significantly more adult worms than ivermectin, achieved a longer suppression in prevalence. These results highlight the importance of considering the macrofilaricidal activity of drugs when designing control programs, because macrofilaricidal activity determines the transmission dynamics after program interruption [35]. Additionally, a small benefit is gained from adding ivermectin to a macrofilaricidal drug, a finding that has implications for current suggestions of strategies based on ivermectin combined with the macrofilaricidal albendazole [35].

### Cost-Effectiveness Analysis

Traditional cost-effectiveness analyses that compare the relative costs and effects of competing health interventions have been conducted for some neglected vector-borne diseases. For example, vector control of Chagas disease through residual spraying was shown to be highly cost-effective in Guatemala [36]. Another study compared the relative cost-effectiveness of different implementation methods (vertical, horizontal, or mixed) to reveal that the mixed strategy was optimal [37]. Extensions regarding the impact of residual spraying on resistance evolution or the impact of different insecticide delivery schemes on vector control are warranted to more fully evaluate long-term consequences of interventions.

Other examples of traditional cost-effectiveness studies are the analyses of drug schemes and of diagnostic/therapeutic strategies that were assessed for visceral leishmaniasis [38], [39]. Using a decision analysis model, the optimal strategy for the identification of diseased individuals and their treatment was evaluated for regions where leishmaniasis is endemic [39]. It was shown that the cost of the drug determines the optimal strategy [39]. Future research could address the impact of resistance evolution or the potential benefit of combined drug interventions [38].

A traditional cost-effectiveness analysis of vector control interventions has also been carried out for dengue in urban areas of Cambodia. Insecticide-based larval control performed twice a year was found to be cost-effective in reducing dengue burden [40]. Interestingly, there was no statistical difference in the effectiveness obtained in areas with two rounds of larval control compared to areas with one round of larval control, implying that potentially less insecticide could be used to prevent dengue in that setting, which would also conserve resources and reduce resistance [41].

## Limitations, Challenges, and Future Directions

We have come a long way since Ronald Ross's early seminal work. However, the increasing availability of complex data poses additional challenges regarding their efficient use by expanding the modeler's horizons into new micro and macro model structures. Recent advances in molecular biology and genetics provide new tools to monitor micro diversity among pathogens and vertebrate and invertebrate hosts. On the other extreme, spatial and social dimensions push the limits of heterogeneities at the macro level. Although macro heterogeneity has received more attention in the past from modelers [42] than micro heterogeneity, current population dynamics paradigms seem ill-equipped to make appropriate predictions in this context and need to be expanded. Formal logical frameworks that explicitly address both data complexities by expanding the notions of efficacy of control measures become necessary since they help in understanding the various contributions of each component to the summary measures of efficacy.

Complex models, where spatial structure, seasonal “forcing”, and/or stochasticity influence the dynamics and the impact of interventions, and where computer simulation needs to be used to generate theory, must be reliable and precise in order to be trusted by the scientific community. Improvements in model sensitivity analysis, validation and diagnostics against independent data, and the availability of alternative model fitting techniques based on Monte Carlo or resampling methods, along with the power of today's computing platforms, are expected to fulfill the demand for formal estimation procedures of confidence intervals for model parameters and predictions [43], [44]. Progress in these areas will help consolidate the partnership between modelers and empiricists, including experts in the disease system of interest, providers of epidemiological data, and those responsible for policy decisions. The title of Joel Cohen's recent paper [45] captures the essence of what the future has reserved in this discipline: “Mathematics Is Biology's Next Microscope, Only Better; Biology Is Mathematics' Next Physics, Only Better”.

Another important future direction is the merger of cost-effectiveness analysis with models of transmission dynamics to address both the short- and long-term impact of resource allocation, while also addressing parameter and model uncertainty [44]–[47]. Traditional cost-effectiveness analyses are generally performed as a one-time comparative analysis of interventions for a cohort of individuals without considering transmission dynamics. However, one-time analyses incorporate neither the dynamic aspect of immunity in a population immunity nor the evolutionary consequences of interventions upon hosts, vectors, and pathogens, which in themselves modify the trade-off between costs and benefits of interventions with time [46], [47].

Indeed, the future directions of modeling pose an interesting challenge. As the field of modeling expands, taking into account biological and ecological details, incorporating dynamic and evolutionary aspects, considering the short- and long-term benefits and consequences, and incorporating uncertainty in parameters and predictions, the need to clearly and properly state a model's results and predictions becomes paramount so that the insights may inform policy.

## Conclusion

Mathematical modeling and cost-effectiveness analysis are essential tools for addressing research questions related to the control of neglected vector-borne diseases. Modelers need to take into consideration a variety of factors, such as pathogen and vector evolution, combined intervention strategies, novel interventions, and the temporal dynamics of disease transmission in order to accurately estimate the benefits and costs of interventions, as well as to predict outcomes.

We have outlined approaches to model parameterization and sensitivity analysis that are fundamental to the interpretation of modeling results. We argue that sensitivity analyses are necessary to handle uncertainty in disease systems, including our incomplete knowledge of “true” parameter values. However, it is important to keep in mind that models should only be as complicated as needed to avoid unnecessary “pseudo-realism” derived from complex models that cannot be parameterized [48]. Communication among modelers, epidemiologists, ecologists, and molecular biologists is essential for the development of realistic models that take into account established knowledge to advance an understanding of systems and to inform public health decisions.

We advocate the use of mathematical models in the analysis of control programs. Several steps in this process are active areas of research, notably the merging of transmission dynamics with cost-effectiveness analysis. As a future trend, we anticipate an increasing partnership between theoretical and field researchers. Such interactions would facilitate the development of a data-driven model that could offer practical guidance to inform policy decisions.

**Box 1. Key Learning Points**

1. The dynamics of an infectious disease in a population and the impact of control measures are not necessarily intuitive because of the dependence structure of transmission of pathogens between individuals through vectors, which gives rise to nonlinear dynamics.
2. Quantitative methods, including mathematical models and cost-effectiveness analysis, can help understand nonlinearities, aid prediction of future dynamics, and allow comparisons among competing control strategies.
3. Control strategies targeting the pathogen, such as drug administration, or the vector, such as insecticide control, can lead to resistance and/or virulence evolution. These factors should be acknowledged in evaluating the long-term impact of control strategies.
4. Increasingly, data on pathogen diversity will become available, allowing for more realistic models. Control measures, such as vaccination or mass drug administration, can change pathogen diversity.
5. Merging transmission dynamics modeling with cost-effectiveness analysis allows for the short- and long-term assessment of optimal control strategies.

**Box 2. Five Key Papers in the Field of “Modeling of Control Interventions for Infectious Diseases”**

1. “Mathematical models of disease transmission” by N. C. Grassly and C. Fraser [2]. An intuitive introduction to the translation of biological process into mathematical models.
2. “Uses and abuses of mathematics in biology” by R. M. May [48]. A quick viewpoint on the decision of being too simple or too complex in the developing of mathematical models.
3. “Evaluating the cost-effectiveness of vaccination programmes: a dynamic perspective” by W. J. Edmunds, G. F. Medley, and D. J. Nokes [46]. A clear presentation of the impact of introducing a dynamic component into “traditional” cost-effectiveness analysis.
4. “Uncertainty and sensitivity analyses of a dynamic economic evaluation model for vaccination programs” by R. J. D. Tebbens et al. [44]. A thorough exploration of the sensitivity analysis methods for mathematical models that aim at evaluating intervention strategies.
5. “Antibiotic and insecticide resistance modeling–is it time to start talking?” by S. L. Peck [7]. An interesting comparison of different modeling approaches into resistance evolution.

## Supporting Information

Alternative Language Abstract S1 Translation of the Abstract into Portuguese by Paula Mendes Luz
(0.03 MB DOC)
