# Supplementary material for: Modeling Transmission Dynamics and Control of Vector-Borne Neglected Tropical Diseases
Source: PLoS Negl Trop Dis. 2010 Oct 26;4(10):e761. doi: 10.1371/journal.pntd.0000761 (PMC2964290; doi:10.1371/journal.pntd.0000761)
Supplement: Alternative Language Abstract S1 — Translation of the Abstract into Portuguese by Paula Mendes Luz (0.03 MB DOC) [file pntd.0000761.s001.doc]

**Abstract (Portuguese version, Word count = 119)**

As doenças tropicais negligenciadas afetam mais de um bilhão de pessoas em todo o mundo. As populações que mais sofrem com essas doenças tipicamente também apresentam maiores restrições orçamentárias. A modelagem matemática da transmissão de doenças e as análises de custo-efetividade podem desempenhar um papel central na tentativa de maximizar os recursos finitos destinados as doenças tropicais negligenciadas. Nós revisamos as contribuições da modelagem matemática na otimização das estratégias de intervenção para doenças tropicais negligenciadas. Nós propomos futuras direções para o campo da modelagem destas doenças que incluem integrar novos conhecimentos a respeito da ecologia do parasita e do vetor, incorporar respostas evolutivas as intervenções e expandir as análises de sensibilidade com o intuito de atingir resultados mais robustos.
